# Splicing Factor DDX23, Transcriptionally Activated by E2F1, Promotes Ovarian Cancer Progression by Regulating FOXM1

**DOI:** 10.3389/fonc.2021.749144

**Published:** 2021-12-13

**Authors:** Chen Zhao, Yingwei Li, Chunping Qiu, Jingying Chen, Huan Wu, Qiuman Wang, Xinyue Ma, Kun Song, Beihua Kong

**Affiliations:** ^1^ Department of Obstetrics and Gynecology, Qilu Hospital, Cheeloo College of Medicine, Shandong University, Jinan, China; ^2^ Gynecology Oncology Key Laboratory, Qilu Hospital, Shandong University, Jinan, China

**Keywords:** ovarian cancer, DDX23, proliferation, invasion, FOXM1, mRNA processing

## Abstract

Ovarian carcinoma remains the most lethal gynecological carcinoma. Abnormal expression of splicing factors is closely related to the occurrence and development of tumors. The DEAD-box RNA helicases are important members of the splicing factor family. However, their role in the occurrence and progression of ovarian cancer is still unclear. In this study, we identified DEAD-box helicase 23 (DDX23) as a key DEAD-box RNA helicase in ovarian cancer using bioinformatics methods. We determined that DDX23 was upregulated in ovarian cancer and its high expression predicted poor prognosis. Functional assays indicated that DDX23 silencing significantly impeded cell proliferation/invasion *in vitro* and tumor growth *in vivo*. Mechanistically, transcriptomic analysis showed that DDX23 was involved in mRNA processing in ovarian cancer cells. Specifically, DDX23 regulated the mRNA processing of FOXM1. DDX23 silencing reduced the production of FOXM1C, the major oncogenic transcript of FOXM1 in ovarian cancer, thereby decreasing the FOXM1 protein expression and attenuating the malignant progression of ovarian cancer. Rescue assays indicated that FOXM1 was a key executor in DDX23-induced malignant phenotype of ovarian cancer. Furthermore, we confirmed that DDX23 was transcriptionally activated by the transcription factor (TF) E2F1 in ovarian cancer using luciferase reporter assays and chromatin immunoprecipitation (ChIP) assays. In conclusion, our study demonstrates that high DDX23 expression is involved in malignant behavior of ovarian cancer and DDX23 may become a potential target for precision therapy of ovarian cancer.

## Introduction

According to statistics from the American Cancer Society (ACS), ovarian cancer is the most lethal gynecological malignancy, ranking fifth among the mortality rates of female cancers (1). Globally, the five-year relative survival rate is generally between 30% and 40% (2). High-grade serous ovarian carcinoma (HGSOC) has the highest incidence and aggressiveness of all subtypes, and accounts for 70-80% of ovarian cancer deaths (3, 4). Current first-line treatments for ovarian cancer include both surgery and systemic treatment. The application of antiangiogenic agents and poly ADP-ribose polymerase (PARP) inhibitors has produced beneficial therapeutic effects for ovarian cancer patients (5, 6). Despite the continued progress in diagnosis and treatment technologies, some patients still relapse in a short time. Therefore, further research is needed to gain new insights into the pathogenesis of ovarian cancer.

mRNA splicing is ubiquitous in human genes. Specifically, the spliceosome removes introns to produce different mature mRNAs, which contribute to the expansion of genomic coding capacity and proteomic diversity (7, 8). Emerging data suggest that aberrant splicing or abnormal expression of splicing factors is associated with cancer progression and cancer immune disorders (9, 10). Many studies have shown that aberrant mRNA splicing is involved in key processes of ovarian cancer development. For example, splicing factor SFPQ participates in *caspase-9* alternative splicing and its overexpression is correlated with platinum resistance (11). Splicing factor SRp20 knockdown impairs growth and malignancy of ovarian cancer cells (12). We have previously shown that splicing factor USP39 and CTNNBL1 were overexpressed in HGSOC and predicted poor clinical outcomes (13, 14).

The RNA helicase family is an important part of splicing factors (15). Members of the DEAD-box RNA helicase family, with conserved sequence Asp-Glu-Ala-Asp (D-E-A-D), play important roles in various aspects of RNA processing, from transcription to RNA decay (16). Therefore, they are given crucial function in tumorigenesis and tumor development. A study has shown that DDX5 is amplified and associated with breast cancer proliferation (17). In addition, DDX39B is overexpressed in colorectal cancer (CRC) and enhances the migration and invasion of CRC cells (18). DDX23 belongs to DEAD-box family of RNA helicases and plays a crucial role in spliceosome formation and pre-mRNA splicing (15). Missense alterations in DDX23 have been reported to be associated with a syndrome characterized by atypical neurodevelopment (19). Abnormal DDX23 expression has been implicated in glioma progression and poor survival (20). However, the specific role of DDX23 in ovarian cancer is less studied.

In this study, DDX23 was first identified as a key DEAD-box RNA helicase in ovarian cancer, and its overexpression was associated with poor clinical outcomes. Functional assays indicated that DDX23 silencing significantly impeded cell proliferation/invasion *in vitro* and tumor growth *in vivo*. Mechanistically, DDX23 regulated the mRNA processing of FOXM1 and DDX23 silencing reduced the production of FOXM1C. FOXM1 was a key executor in DDX23-induced malignant phenotype of ovarian cancer. Moreover, DDX23 was transcriptionally activated by the E2F1 in ovarian cancer. Taken together, this study demonstrates the clinical and biological significance of DDX23 in ovarian cancer and provides a new target for tumor precision therapy.

## Materials And Methods

### Bioinformatics Analysis

The genes involved in mRNA splicing (major pathway) were obtained from GeneCards (https://pathcards.genecards.org/card/mrna_splicing_-_major_pathway) (21). The protein expression data were obtained from the clinical proteomic tumor analysis consortium (CPTAC) (https://cptac-data-portal.georgetown.edu/studies) (22). The Cancer Genome Atlas (TCGA) ovarian cancer data (AffyU133a, n = 593) were obtained from UCSC Xena (http://xena.ucsc.edu/) (23). Survival curves were plotted by Kaplan-Meier plotter (https://kmplot.com/analysis/) (24). Co-expression analysis was performed on cbioportal (https://www.cbioportal.org/) (25). JASPAR (http://jaspar.genereg.net/) (26) and Cistrome Cancer (http://cistrome.org/CistromeCancer/) (27) were used to predict potential TFs. Visualization and analysis of TFs binding peaks were performed with the use of Cistrome Data Browser (http://cistrome.org/db/#/) (28) based on the online chromatin immunoprecipitation sequencing (ChIP-seq) data. Gene Ontology (GO) analysis was conducted on WebGestalt (http://www.webgestalt.org/) (29). The TCGA differential expression gene (DEG) list of ovarian cancer was obtained from Gene Expression Profiling Interactive Analysis (GEPIA) (http://gepia.cancer-pku.cn/) (30).

### Tissue Samples and Clinical Information

Ovarian cancer specimens were obtained from primary patients without neoadjuvant chemotherapy. Fallopian tube (FT) specimens from patients with benign diseases were used as controls. The 46 fresh-frozen ovarian cancer tissues and 29 FT tissues were obtained for quantitative real-time PCR (qRT-PCR) analysis. A total of 124 ovarian cancer and 69 FT specimens from our center were used for clinical information analysis. Patients’ informed consent were provided. The study had been approved by the Ethics Committee of Shandong University.

### Immunohistochemistry Staining

The fresh tissues were formalin fixed and paraffin embedded. An immunohistochemistry (IHC) staining kit (ZSGB-BIO, China) was used for staining of tissue microarray (TMA) sections or xenografts tissue sections following the manufacturer’s instructions. Paraffin sections were deparaffined with xylene and rehydrated with ethanol. After antigen retrieval, 3% hydrogen peroxide and goat serum were used to block the endogenous peroxidase and nonspecific binding respectively. Tissue sections were incubated with primary antibodies anti-DDX23 (ab70459, Abcam) and anti-Ki-67 (#9449, CST) at 4°C overnight. The next day, tissue was labeled with secondary antibody and detected using the diaminobenzidine (DAB) staining system.

Two pathologists completed the IHC staining score independently. The intensity of staining was scored as 0 (negative), 1 (weak), 2 (moderate), or 3 (strong). The final H-score (0-300) was determined by the extent and intensity of staining (H-score = percentage of weak intensity area×1+ percentage of moderate intensity area×2+ percentage of strong intensity area×3). The specimens were divided into high expression group (final score < 170) and low expression group (final score ≥ 170).

### RNA Isolation and qRT-PCR

TRIzol reagent (Invitrogen, USA) was used for total RNA extraction. PrimeScript RT Reagent Kit (Takara, Japan) and SYBR-Green qPCR master mix (Takara, Japan) were used for RNA reverse transcription and qRT-PCR respectively. ACTB served as an internal control. The primers used are listed in **Supplementary Table 1**.

### Cell Lines and Cell Culture

A2780 and SKOV3 were cultured in RPMI 1640 medium plus 10% fetal bovine serum (FBS) (BioInd, Israel). HEY and HEK293T were cultured in DMEM medium plus 10% FBS. Cells were cultured in standard conditions (37°C, 5% CO2) in a humidified incubator.

### Plasmid Constructs and Cell Transfection

The shDDX23 sequence was cloned into pLKO.1 vector (Addgene, United States). The open reading frames (ORFs) of E2F1 and FOXM1 were cloned into pLenti-C-Myc-DDK-IRES-Puro (PCMV) vector (Origene, USA) separately. The psPAX2, pMD2.G and constructed lentivirus vectors were co-transfected into HEK293T cells for lentivirus production. To gain stable-expression, ovarian cancer cells were infected with lentivirus for 24 hours and selected for 7 days in a medium including puromycin (2 μg/mL, Merck Millipore, USA).

The small interfering RNAs (siRNAs) targeting DDX23, E2F1, FOXM1 were obtained from GenePharma (Shanghai, China). Transient transfection was carried out by Lipofectamine 2000 reagent (Invitrogen, USA) following the manufacturer’s instructions. Details of shRNA or siRNA sequences are shown in **Supplementary Table 1**.

### Cell Proliferation Assay

The 3-(4, 5)-dimethylthiahiazo(-z-y1)-3,5-di-phenytetra-zoliumromide (MTT) assay was conducted to measure cell proliferation ability. Cells (800–1000 cells/well) were seeded in 96-well plates, then incubated and monitored continuously. At a fixed time point of each day, 20 μL 5 mg/mL of MTT (Sigma-Aldrich, USA) solution was added to each well. After 4 hours of incubation, the supernatant was replaced by 100 μL DMSO (Sangon Biotech, China). The absorbance value at 490nm was quantified by a microplate reader (Bio-Rad, USA).

### Clonogenic Assay

Cells (800–1000 cells/well) were cultured in 6-well plates under standard condition for 2 weeks. Methanol was applied for colony fixation and 0.1% crystal violet was applied for staining. Colonies containing more than 50 cells were included in statistical analysis.

### Cell Cycle Assay

Flow cytometry was used to analyze cell cycle progression. Each group of ovarian cancer cells was harvested and stained with propidium iodide (PI) according to the manufacturer’s protocol (MultiSciences, China). The cell cycle distribution was analyzed by A Modifit LT software (BD Biosciences, USA).

### Western Blotting

RIPA Lysis Buffer (Beyotime, China), supplemented with 1% PMSF, was used for cell lysis. A BCA Assay Kit (Millipore, USA) was used to quantify the protein concentration. Protein samples were separated by SDS-PAGE and transferred to PVDF membranes (Millipore, USA), and then blocked in 5% skimmed milk for 1 hour. The membranes were incubated in diluted primary antibodies at 4°C overnight. The target proteins were labeled with HRP-conjugated secondary antibodies and detected with an ECL system (PerkinElmer, USA). β-actin was used as an endogenous control. All antibodies are listed in **Supplementary Table 2**.

### Cell Migration and Invasion Assays

Cells (1×10^5^) suspended in 200 μL serum-free medium were seeded into the upper Transwell chambers (8μm pores, BD Biosciences, USA). The lower compartments contained 700 μL medium with 20% FBS. Methanol was applied for cell fixation and 0.1% crystal violet was applied for staining. Cells that penetrated through the chambers were counted under a light microscope.

Cell motility ability was also evaluated by wound healing assays. Ovarian cancer cells were cultured in 24-well plates for appropriate time. Then straight scratches were produced on the confluent cell monolayer with 20 μL pipette tips. The scratch width was measured at appropriate time points (0 h, 12 h) after scratching.

### Nude Mouse Xenograft Models

Female BALB/c nude mice (aged 4–5 weeks, NBRI of Nanjing University, China) were randomly divided into two groups and injected subcutaneously with DDX23 knockdown or control cells (HEY, 5×10^6^). Mice were kept in the SFP environment before they were euthanized. Then tumors were harvested and weighed. Animal experiments were approved by Shandong University Animal Care and Use Committee.

### Luciferase Reporter Assay

HEK293T cells were co-transfected with DDX23 wild-type (WT) or mutant-type (MT) (Deletion mutation) promoter reporter vectors, PCMV-NC or PCMV-E2F1 and pRL-TK plasmids using Lipofectamine 2000. After 48 hours of transfection, the luciferase activity was tested by Dual-Glo Luciferase Assay System (Promega, USA). The relative luciferase activity was determined by the ratio of firefly luminescence to Renilla luminescence.

### Chromatin Immunoprecipitation Assay

A Chromatin immunoprecipitation kit (Beyotime, China) was used for ChIP assay as previously described (13). E2F1 antibody and IgG rabbit antibody were obtained from Cell Signaling Technology (CST, USA) (**Supplementary Table 2**). Reverse transcription PCR was performed to analyze the purified DNA. The primer sequences for DDX23 promoter are listed in **Supplementary Table 1**.

### RNA Sequencing and Differential Gene Expression Analysis

Total RNA was extracted from A2780 cells of DDX23 knockdown and control group with Trizol reagent. Then high-throughput RNA sequencing (RNA-seq) assay was performed by the Biomaker Technologies (Beijing, China). The threshold for different expression was set to 1.5-fold change (FC) and *P* < 0.05 was the significance threshold.

### Statistical Analysis

SPSS statistics 24.0 and GraphPad Prism 8.0 were used in data analysis. The chi-square test and student’s t test were used to analyze statistically significant differences between groups. Univariate and multivariate Cox proportional hazard regression analysis was used to analyze high-risk factors related to overall survival (OS). The survival curves of independent high-risk factors were plotted using Kaplan-Meier analysis. The data of three independent experiments were presented as the means ± SEMs. *P* < 0.05 was considered statistically significant.

## Results

### DDX23 Was Upregulated in Ovarian Cancer and Associated With Poor Clinical Outcomes

**Figure 1:**
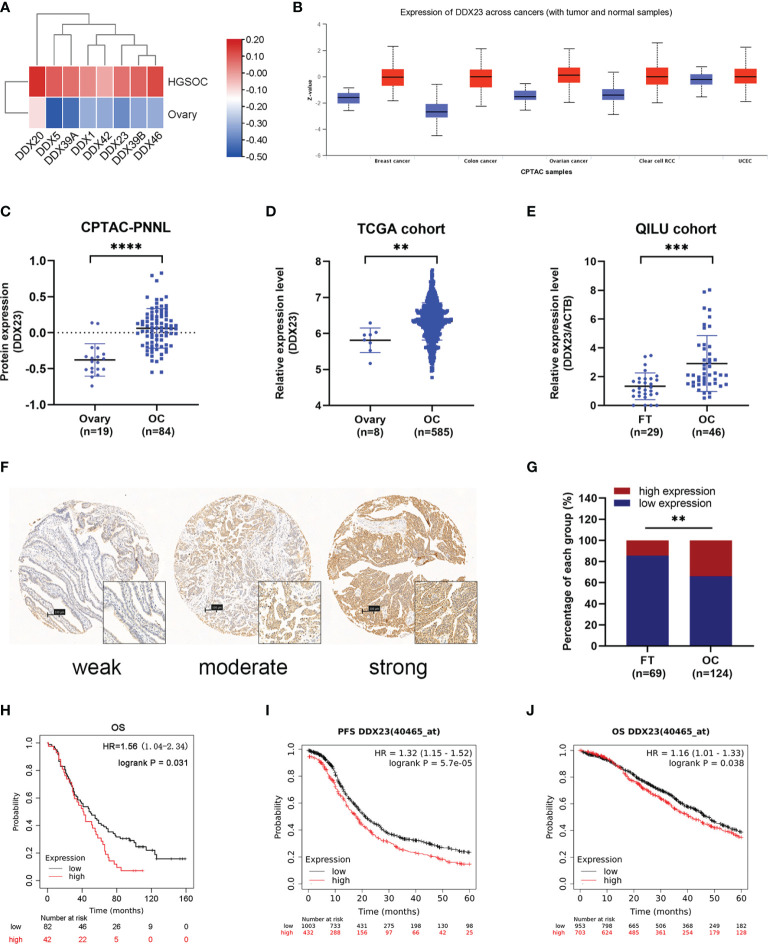
DDX23 was upregulated in ovarian cancer and associated with poor clinical outcomes. **(A)** Heatmap map reflecting the expression of 8 DEAD-box RNA helicases involved in the mRNA splicing in HGSOC and normal ovary samples based on CPTAC proteomic data. **(B)** DDX23 protein expression profile in various cancers based on CPTAC proteomic data. **(C)** DDX23 protein expression in ovarian cancer and normal ovary samples in CPTAC-PNNL cohort. **(D)** DDX23 mRNA expression in ovarian cancer and normal ovary samples in a TCGA cohort (AffyU133a, n = 593). **(E)** qRT-PCR analysis of DDX23 mRNA expression in 46 HGSOC and 29 FT tissue samples in Qilu cohort. **(F)** Representative IHC staining images of DDX23 in FT and ovarian cancer tissues based on TMAs. **(G)** Statistical analysis of the DDX23 expression profile in 69 FT and 124 ovarian cancer tissues based on IHC staining score of TMAs. **(H)** Kaplan-Meier analysis of the correlation between DDX23 expression and OS based on the follow-up information from our TMAs. **(I, J)** Kaplan-Meier analysis of PFS **(I)** and OS **(J)** in ovarian cancer patients with high- or low- DDX23 expression based on data from Kaplan-Meier Plotter. HGSOC, high-grade serous ovarian carcinoma; CPTAC, clinical proteomic tumor analysis consortium; PNNL, Pacific Northwest National Laboratory; TCGA, The Cancer Genome Atlas; FT, fallopian tube; IHC, immunohistochemistry; TMAs, tissue microarrays; OS, overall survival; PFS, progression-free survival. **P < 0.01, ***P < 0.001, ****P < 0.0001.

To clarify the importance of DEAD-box RNA helicases involved in mRNA processing, we screened 8 candidates of 322 genes related to mRNA splicing (Major Pathway) from the GeneCards online database. Based on CPTAC proteomic data, we found that all 8 genes were upregulated in ovarian cancer (**Figure 1A**). Meanwhile, according to previous transcriptome analysis results, 33 upregulated core splicing factors, including DDX23, were found in HGSOCs (n=6) compared with FT tissues (n=6) (GSE135886) (13). Combined data mining with previous transcriptome analysis results, DDX23 was selected for further investigation. We first investigated its protein expression patterns in various cancer types using CPTAC database. Specifically, DDX23 expression was found to be elevated in multiple tumor types including ovarian cancer (*P* < 0.0001) (**Figures 1B, C**). We further analyzed data of TCGA cohort and found a significantly higher mRNA level of DDX23 in ovarian cancer samples (n = 585) compared with normal ovary samples (n = 8) (*P* < 0.01) (**Figure 1D**). Similarly, we detected the DDX23 mRNA expression in our cohort by qRT-PCR and found that DDX23 had higher expression in ovarian cancer samples (n = 46) than in FT specimens (n = 29) (*P* < 0.001) (**Figure 1E**).

To further explore the relationship between the expression of DDX23 and the clinicopathological characteristics of ovarian cancer patients, IHC analysis was performed using TMAs containing 124 ovarian cancer and 69 FT specimens. Results showed that 14.5% (10/69) FT samples had high DDX23 expression, whereas 33.9% (42/124) ovarian cancer samples belonged to high DDX23 group. Compared with FT specimens, IHC staining revealed significantly higher DDX23 expression in ovarian cancer specimens (*P* < 0.01) (**Figures 1F, G**). Clinicopathological feature analysis indicated that high DDX23 expression was positively correlated with poor OS (P = 0.037) (**Table 1**). In addition, univariate and multivariate Cox proportional hazard regression analysis indicated that DDX23 expression was an independent high-risk factor for OS (hazard ratio [HR] 1.58, 95% confidence interval [CI] 1.05–2.37, P = 0.029), besides FIGO stage (HR 2.00, 95% CI 1.19–3.37, P = 0.009) (**Table 2**). We further performed Kaplan-Meier survival analysis in our cohort, and confirmed that patients in the high DDX23 expression group had a shorter OS than those in the low expression group (HR 1.56, 95% CI 1.04–2.34, P = 0.031) (**Figure 1H**). Meanwhile, based on the online data of the Kaplan-Meier plotter, we also verified that patients with high DDX23 expression had significantly worse progression-free survival (PFS) (HR 1.32, 95% CI 1.15–1.52, P = 5.7e-0.5) and OS (HR 1.16, 95% CI 1.01–1.33, P = 0.038) rates than patients with low expression (**Figures 1I, J**). Taken together, these results strongly indicated that DDX23 was highly expressed in ovarian cancer tissues and was significantly associated with poor prognosis in ovarian cancer patients.

**Table 1:** Correlation of clinical characteristics with DDX23 expression.

Clinical characteristics		DDX23 expression			*P* value
		Total (n = 124)	Low expression (n = 82)	High expression (n = 42)	
**Age (years)**	<56	55 (44.4)	37 (45.1)	18 (42.9)	0.810
	≥56	69 (55.6)	45 (54.9)	24 (57.1)	
**FIGO stage (2014)**	I and II	26 (21.0)	17 (20.7)	9 (21.4)	0.928
	II and III	98 (79.0)	65 (79.3)	33 (78.6)	
**Histology**	HGSOC	103 (83.1)	68 (82.9)	35 (83.3)	0.404
	Non-HGSOC	8 (6.5)	6 (7.3)	2 (4.8)	
	Unknown	13 (10.5)	8 (9.8)	5 (11.9)	
**Grade**	II (moderately)	8 (6.5)	6 (7.3)	2 (4.8)	0.859
	III (poorly)	110 (88.7)	72 (87.8)	38 (90.5)	
	Unknown	6 (4.8)	4 (4.9)	2 (4.8)	
**Ascites**	Yes	23 (18.5)	14 (17.1)	9 (21.4)	0.706
	No	7 (5.6)	4 (4.9)	3 (7.1)	
	Unknown	94 (75.8)	64 (78.0)	30 (71.4)	
**CA-125 (U/mL)**	<785	58 (46.8)	36 (43.9)	22 (52.4)	0.371
	≥785	66 (53.2)	46 (56.1)	20 (47.6)	
**Tumor diameter (cm)**	<8	37 (29.8)	27 (32.9)	10 (23.8)	0.294
	≥8	87 (70.2)	55 (67.1)	32 (76.2)	
**Residual disease (cm)**	<1	51 (41.1)	38 (46.3)	13 (31.0)	0.099
	≥1	73 (58.9)	44 (53.7)	29 (69.0)	
**Adjuvant chemotherapy**	Yes	122 (98.4)	81 (98.8)	41 (97.6)	1.000
	No	2 (1.6)	1 (1.2)	1 (2.4)	
**Death**	Yes	103 (83.1)	64 (78.0)	39 (92.9)	**0.037**
	No	21 (16.9)	18 (22.0)	3 (7.1)	
**Follow-up time (month)**		45.5 (1-159)	49 (1-159)	41.5 (1-110)	0.166

Values are present as n (%) or median (range). DDX23, DEAD-Box Helicase 23; FIGO, International Federation of Gynecology and Obstetrics; HGSOC, high-grade serous ovarian carcinoma; CA-125, Cancer Antigen 125.

**Table 2:** Univariate and multivariate Cox proportional hazards regression analysis of OS.

Clinical characteristics		Univariate		Multivariate	
		HR (95%CI)	*P* value	HR (95%CI)	*P* value
**DDX23 expression**	Low	1	0.034	1	0.029
	High	1.56 (1.04-2.34)		1.58 (1.05-2.37)	
**Age (years)**	<56	1	0.616		
	≥56	1.01 (0.99-1.03)			
**FIGO stage (2014)**	I and II	1	0.010	1	0.009
	II and III	1.98 (1.17-3.34)		2.00 (1.19-3.37)	
**Histology**	HGSOC	1			
	Non-HGSOC	1.14 (0.53-2.47)	0.736		
	Unknown	1.49 (0.81-2.74)	0.196		
**Grade**	II (moderately)	0.75 (0.25-2.25)	0.609		
	III (poorly)	0.67 (0.29-1.55)	0.349		
	Unknown	1			
**Ascites**	Yes	0.90 (0.56-1.46)	0.681		
	No	0.46 (0.16-1.34)	0.153		
	Unknown	1			
**CA-125 (U/mL)**	<785	1	0.242		
	≥785	1.26 (0.86-1.86)			
**Tumor diameter (cm)**	<8	1	0.924		
	≥8	0.98 (0.65-1.49)			
**Residual disease (cm)**	<1	1	0.008		
	≥1	1.73 (1.15-2.59)			
**Adjuvant chemotherapy**	Yes	0.92 (0.23-3.73)	0.903		
	No	1			

OS, overall survival; DDX23, DEAD-Box Helicase 23; FIGO, International Federation of Gynecology and Obstetrics; CA-125, Cancer Antigen 125.

### DDX23 Was Required for the Proliferation and Cell Cycle Progression of Ovarian Cancer Cells

**Figure 2:**
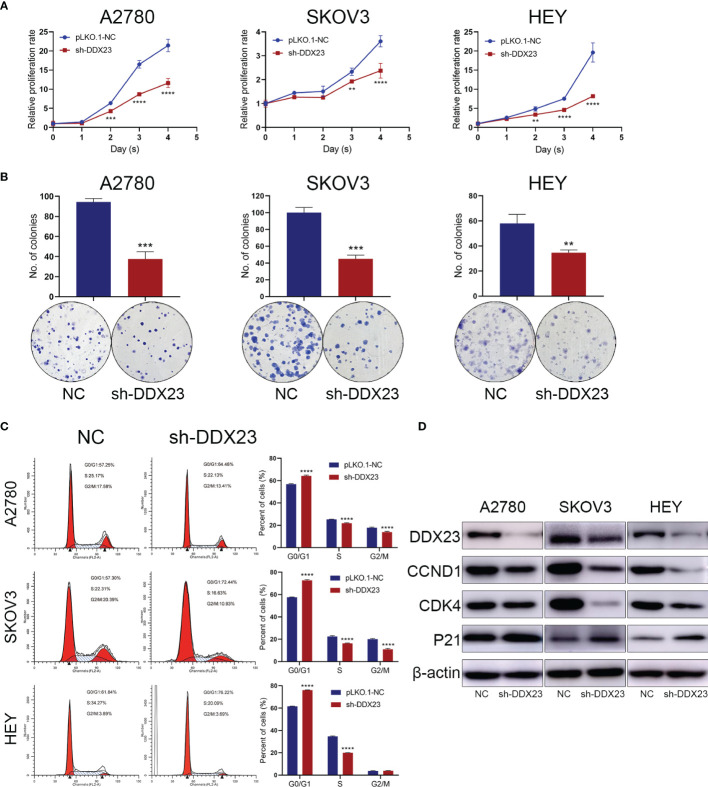
DDX23 was required for the proliferation and cell cycle progression of ovarian cancer cells. **(A, B)** Representative MTT proliferation **(A)** and clonogenic **(B)** assays in A2780, SKOV3 and HEY cells with or without DDX23 knockdown. **(C)** Cell cycle analysis of A2780, SKOV3, and HEY cells with or without DDX23 knockdown was performed by flow cytometry (left). Graphs depict the distribution of cells in indicated phases of the cell cycle (right). **(D)** Western blotting analysis of cell cycle regulatory proteins in A2780, SKOV3, and HEY cells transfected with sh-NC or sh-DDX23. Data are presented as mean ± SEM. **P < 0.01, ***P < 0.001, ****P < 0.0001.

Given that DDX23 was upregulated in ovarian cancer, we then explored the role of DDX23 in the proliferation of ovarian cancer cells. First, DDX23 knockdown ovarian cell lines were established by lentiviral infection. In MTT assays, compared to the negative control (NC) groups, DDX23 silencing inhibited the growth of A2780, SKOV3, and HEY cells and the inhibition was most evident in the last two days (**Figure 2A**). Similarly, in clonogenic assays, DDX23 knockdown reduced the colony formation ability of A2780, SKOV3, and HEY cells by 60% (*P* < 0.001), 55% (*P* < 0.001), and 40% (*P* < 0.01) respectively (**Figure 2B**). To further investigate the effect of DDX23 on cell cycle progression, flow cytometry was conducted. Cell cycle analysis revealed that compared to NC group, DDX23 silencing could increase the percentage of cells in the G1 phase while decreasing the percentage of cells in S phase in three ovarian cancer cell lines (**Figure 2C**). Furthermore, we measured G1 phase arrest related markers by western blotting and the results showed that DDX23 knockdown decreased the expression of CCND1 and CDK4, but increased p21 expression (**Figure 2D**).

Overall, these data suggested that DDX23 was required for ovarian cancer cell proliferation, and DDX23 knockdown inhibited cell proliferation through G1 phase arrest.

### DDX23 Silencing Suppressed the Migration and Invasion of Ovarian Cancer Cells

**Figure 3:**
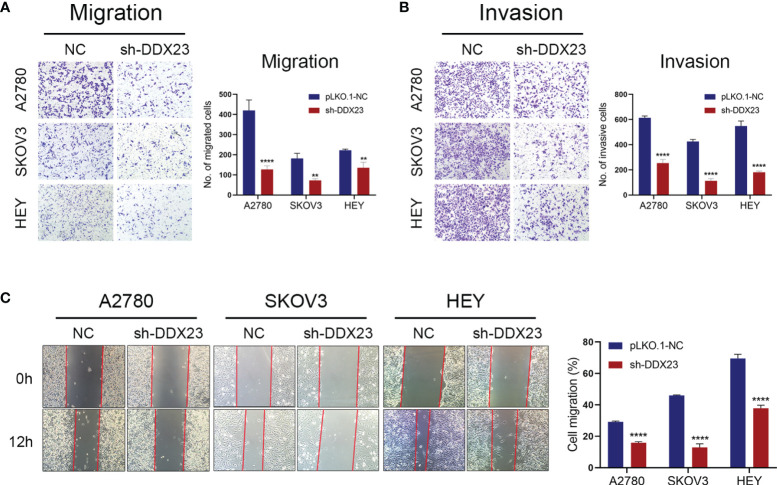
DDX23 silencing suppressed the migration and invasion of ovarian cancer cells. **(A, B)** Representative microscopic images (×10) of A2780, SKOV3, and HEY cells that penetrated through the Transwell chambers in migration **(A)** and invasion **(B)** assays. **(C)** Representative wound healing assays in A2780, SKOV3, and HEY cells with or without DDX23 knockdown. Data are presented as mean ± SEM. **P < 0.01, ****P < 0.0001.

Transwell assays were carried out to detect the effect of DDX23 on the migration and invasion of ovarian cancer cells. Compared to the NC group, DDX23 knockdown could weaken the migration (all *P* < 0.01) and invasion (all *P* < 0.0001) capacity in A2780, SKOV3, and HEY cells (**Figures 3A, B**). In wound healing assays, at 12h post-scratch, ovarian cancer cells with DDX23 knockdown migrated less than NC group in A2780, SKOV3, and HEY cells (all *P* < 0.0001) (**Figure 3C**). These experimental results collectively suggested that DDX23 could promote the migration and invasion ability of ovarian cancer cells.

### DDX23 Knockdown Inhibited the Growth of Xenograft Tumors *in Vivo*

**Figure 4:**
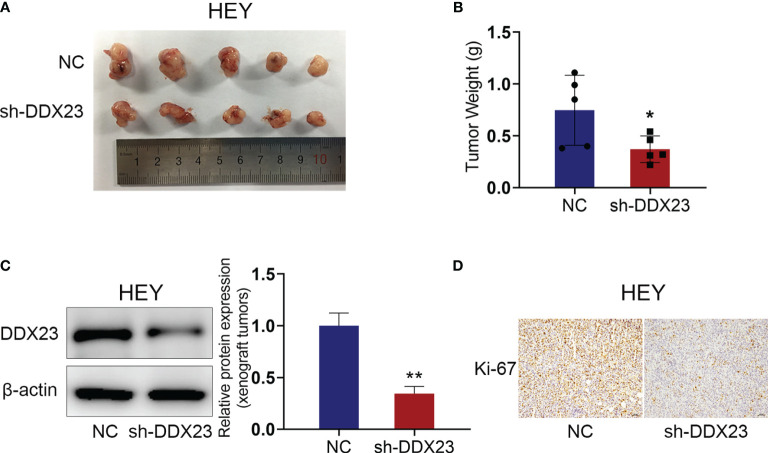
DDX23 knockdown inhibited the growth of xenograft tumors *in vivo*. **(A)** Images of xenograft tumors from mice subcutaneously injected with DDX23 knockdown or control HEY cells (n = 5 mice per group). **(B)** The xenografts tumors were weighed and compared. ***(*C*)*** Western blotting analysis of protein samples extracted from xenografts tumors in DDX23 knockdown or corresponding control group. **(D)** Representative IHC staining patterns of Ki-67 in xenografts tumors in DDX23 knockdown or corresponding control group. IHC, immunohistochemistry. Data are presented as mean ± SEM. *P < 0.05, **P < 0.01.

Since the effect of DDX23 on the progression of ovarian cancer was determined *in vitro*, we further constructed nude mouse xenograft models to explore the role of DDX23 in ovarian cancer tumorigenesis *in vivo*. HEY cells with DDX23 knockdown and the control cells were subcutaneously injected into two groups of nude mice (n=5). As expected, DDX23 silencing could apparently inhibit the growth of xenograft tumors (**Figure 4A**). The tumor weights of NC group were significantly higher than those of DDX23 knockdown group (*P* < 0.05) (**Figure 4B**). The protein expression level in xenograft tumors was also measured to confirm that DDX23 was effectively depleted in the shDDX23 group (*P* < 0.01) (**Figure 4C**). IHC staining showed that Ki-67 expression was decreased in xenograft tumors of shDDX23-treated mice group, indicating that DDX23 knockdown reduced the proliferation activity of tumor cells *in vivo* (**Figure 4D**).

### E2F1 Activated DDX23 Transcription in Ovarian Cancer Cells

**Figure 5:**
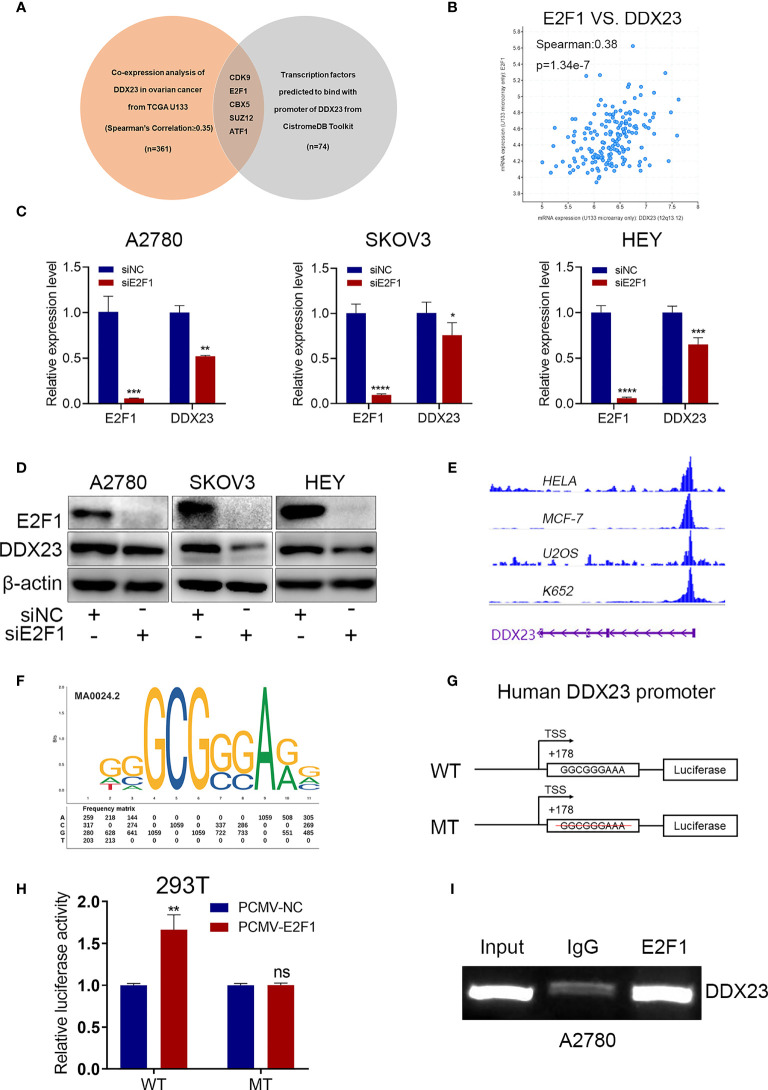
E2F1 activated DDX23 transcription in ovarian cancer cells. **(A)** Venn diagram of 5 hub TFs positively related to DDX23 expression (TCGA U133 microarray, Spearman’s Correlation ≥ 0.35) and predicted to bind with the promoter of DDX23. **(B)** Co-expression analysis between DDX23 and E2F1 expression in ovarian cancer based on the cBioPortal database. **(C, D)** The mRNA and protein levels of E2F1 and DDX23 in ovarian cancer cells with or without E2F1 knockdown were measured by qRT-PCR **(C)** and Western blotting **(D)**. **(E)** Visualization of E2F1 binding peaks. The binding peaks of E2F1 were enriched in the promoter region of DDX23 in HELA, MCF-7, U2OS, and K652 cell lines based on ChIP-seq data from the Cistrome Data Brower. **(F)** The sequence logo of a potential E2F1 binding site on DDX23 promoter predicted by JASPAR**. (G)** Schematic diagram of the DDX23 WT and MT promoter sequences. **(H)** Dual-luciferase reporter assays showing that E2F1 overexpression increased the luciferase activity in HEK293T cells transfected with the DDX23 promoter WT plasmid, but not in cells with MT plasmid. **(I)** ChIP assay and semi-quantitative PCR analysis showed that E2F1 could bind to the DDX23 promoter region directly. TFs, transcription factors; ChIP, Chromatin immunoprecipitation; WT, wild type; MT, mutant type. Data are presented as mean ± SEM. ns, no significant, *P < 0.05, **P < 0.01, ***P < 0.001, ****P < 0.0001.

Dysregulation of TFs is associated with tumor progression. To explore the transcriptional regulatory mechanism of DDX23 expression, we performed co-expression analysis using cBioPortal database to obtain the genes positively related to DDX23 expression (TCGA U133 microarray, Spearman’s Correlation ≥ 0.35) (**Supplementary Table 3**). We also analyzed the TFs predicted to bind to the promoter of DDX23 from Cistrome Data Browser (**Supplementary Table 4**). Subsequently, 5 candidate TFs were screened by determining the intersection of the above two gene sets (**Figure 5A**). The differential expression analysis of the 5 TFs were performed using TCGA-GTEx data, and the results showed that the expression of E2F1 in ovarian cancer increased most significantly compared to the other 4 TFs (**Supplementary Figure S1**). Co-expression analysis revealed that the mRNA expression of E2F1 and DDX23 were positively correlated in ovarian cancer (Spearman’s correlation = 0.38, *P* = 1.34e-7) (**Figure 5B**). To determine whether E2F1 was involved in the regulation of DDX23 transcription, we first detected the expression of DDX23 in E2F1 knockdown and control ovarian cancer cells. We noted that the inhibition of E2F1 by siRNA decreased the expression of DDX23 at both mRNA and protein levels (**Figures 5C, D**). Meanwhile, we found that DDX23 knockdown had no effect on E2F1 expression at both mRNA and protein levels in three ovarian cancer cell lines (**Supplementary Figures S2A, B**).

According to the ChIP-seq data from the Cistrome Data Browser database, we found that the binding peaks of E2F1 were enriched in the promoter region of DDX23 in HELA, MCF-7, U2OS, and K652 cell lines (**Figure 5E**). We next searched the JASPAR online database and a potential E2F1 binding site with the highest score was found on the DDX23 promoter region (**Figure 5F**) (**Supplementary Table 5**). We constructed WT and MT plasmids of DDX23 promoter using pGL4.26 vector (**Figure 5G**). Luciferase assays showed that E2F1 overexpression increased the luciferase activity in HEK293T cells transfected with the DDX23 promoter WT plasmid, but not in cells with MT plasmid. (**Figure 5H**). Subsequently, ChIP assays were performed in A2780 cells to further verify the binding of E2F1 to DDX23 promotor. Results confirmed that E2F1 could bind to the DDX23 promoter region directly (**Figure 5I**). In summary, these data indicated that DDX23 was a direct transcriptional target of E2F1 in ovarian cancer cells.

### Identification of Differentially Expressed Genes Involved in DDX23 Function by RNA-seq

**Figure 6:**
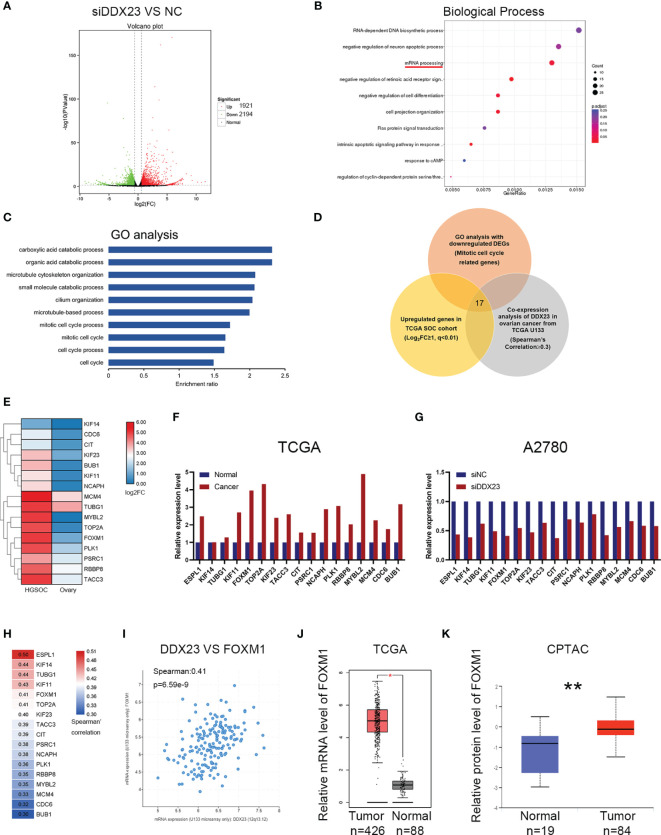
Identification of differentially expressed genes involved in DDX23 function by RNA-seq. **(A)** Volcano plot for the DEGs between siDDX23 and NC groups (1.5-FC, *P* < 0.05). **(B)** Biological process analysis showed that DDX23 was involved in mRNA processing. **(C)** GO enrichment analysis of the downregulated DEGs between siDDX23 and NC groups. **(D)** Venn diagram of 17 hub genes obtained by overlapping three gene sets. Gene set 1, 123 downregulated genes related to mitotic cell cycle processes identified by RNA-seq; Gene set 2, upregulated genes in ovarian cancer from GEPIA (Log_2_FC ≥ 1, q < 0.01); Gene set 3, genes that positively related to DDX23 expression (TCGA U133 microarray, Spearman’s Correlation ≥ 0.3). **(E, F)** Relative mRNA expression of 17 candidate downstream genes in TCGA database. **(G)** qRT-PCR analysis of mRNA expression of 17 candidates in A2780 cells. **(H)** Co-expression analysis between DDX23 and 17candidates expression in ovarian cancer based on the cBioPortal database. **(I)** Co-expression analysis between DDX23 and FOXM1 expression in ovarian cancer based on the cBioPortal database. **(J)** FOXM1 mRNA expression in ovarian cancer and normal ovary samples in TCGA cohort from GEPIA. **(K)** FOXM1 protein expression in ovarian cancer and normal ovary samples in CPTAC cohort. DEG, differential expression gene; NC, negative control; FC, fold change; GO, Gene Ontology; GEPIA, Gene Expression Profiling Interactive Analysis; TCGA, The Cancer Genome Atlas. *P < 0.05, **P < 0.01.

To explore the regulatory mechanisms of DDX23 on tumor progression, RNA‐seq was performed in DDX23 knockdown and control A2780 cells. The changes in the transcriptome with DDX23 knockdown were analyzed and a total of 4115 differentially expressed genes (DEGs) were identified (1.5-FC, *P* < 0.05). There were 1921 upregulated genes and 2194 downregulated genes (**Figure 6A**). Biological process analysis showed that DDX23 was involved in mRNA processing, which confirmed the splicing-related functions of DDX23 in ovarian cancer (**Figure 6B**). Then, GO enrichment analysis was performed with the 2194 downregulated DEGs. Multiple downregulated genes (Gene set 1) were found to be associated with mitotic cell cycle process, which is an important underlying mechanism of tumor progression and consistent with the results of functional assays *in vivo* and *in vitro* (**Figure 6C**). Next, we searched the GEPIA database to obtain the upregulated genes in TCGA ovarian cancer cohort (Log_2_FC ≥ 1, q < 0.01) (Gene set 2). The cBioPortal database was also used to obtain genes that were positively associated with DDX23 expression (TCGA U133 microarray, Spearman’s Correlation ≥ 0.3) (Gene set 3). Finally, 17 genes were screened by overlapping the three gene sets (**Figure 6D**). The expression details of the 17 candidates in TCGA cohort were showed in **Figures 6E, F**. Meanwhile, we demonstrated the low mRNA expression of these 17 genes in DDX23 knockdown A2780 cell line (**Figure 6G**). Co-expression analysis revealed the expression correlation between DDX23 and 17 candidates, and the details are shown in **Figure 6H**. Based on data from Cistrome Cancer, we found that among the five genes (ESPL1, KIF14, TUBG1, KIF11, FOXM1) with the highest correlation with DDX23 expression, four genes (ESPL1, KIF14, TUBG1, KIF11) were potential target genes of FOXM1 (Regular potential score, 0.943365, 0.96575, 0.644621, 0.988351, respectively) (**Supplementary Table 6**). DDX23 was shown to be positively associated with the expression of a well-known oncogene FOXM1 (Spearman’s correlation = 0.41, *P* = 6.59e-9) (**Figure 6I**). Based on data from TCGA and CPTAC, we found that FOXM1 was upregulated in ovarian cancer at both mRNA and protein levels (**Figures 6J, K**). Numerous studies had proven that FOXM1 promoted the progression of various cancer types (31–33). Integrated genomic analyses of ovarian carcinoma from TCGA Research Network reported that the FOXM1 transcription factor network changed significantly in 87% of ovarian cancer cases (34). FOXM1, as a transcription factor, regulated many important proliferation-related target genes (AURB, CCNB1, BIRC5, CDC25, and PLK1, etc.) and was important oncogenic driver in ovarian cancer progression (34–36). Therefore, our subsequent studies focused on FOXM1 as a downstream target of DDX23.

### DDX23 Regulated the Production of the Main Oncogenic Transcript of FOXM1

**Figure 7:**
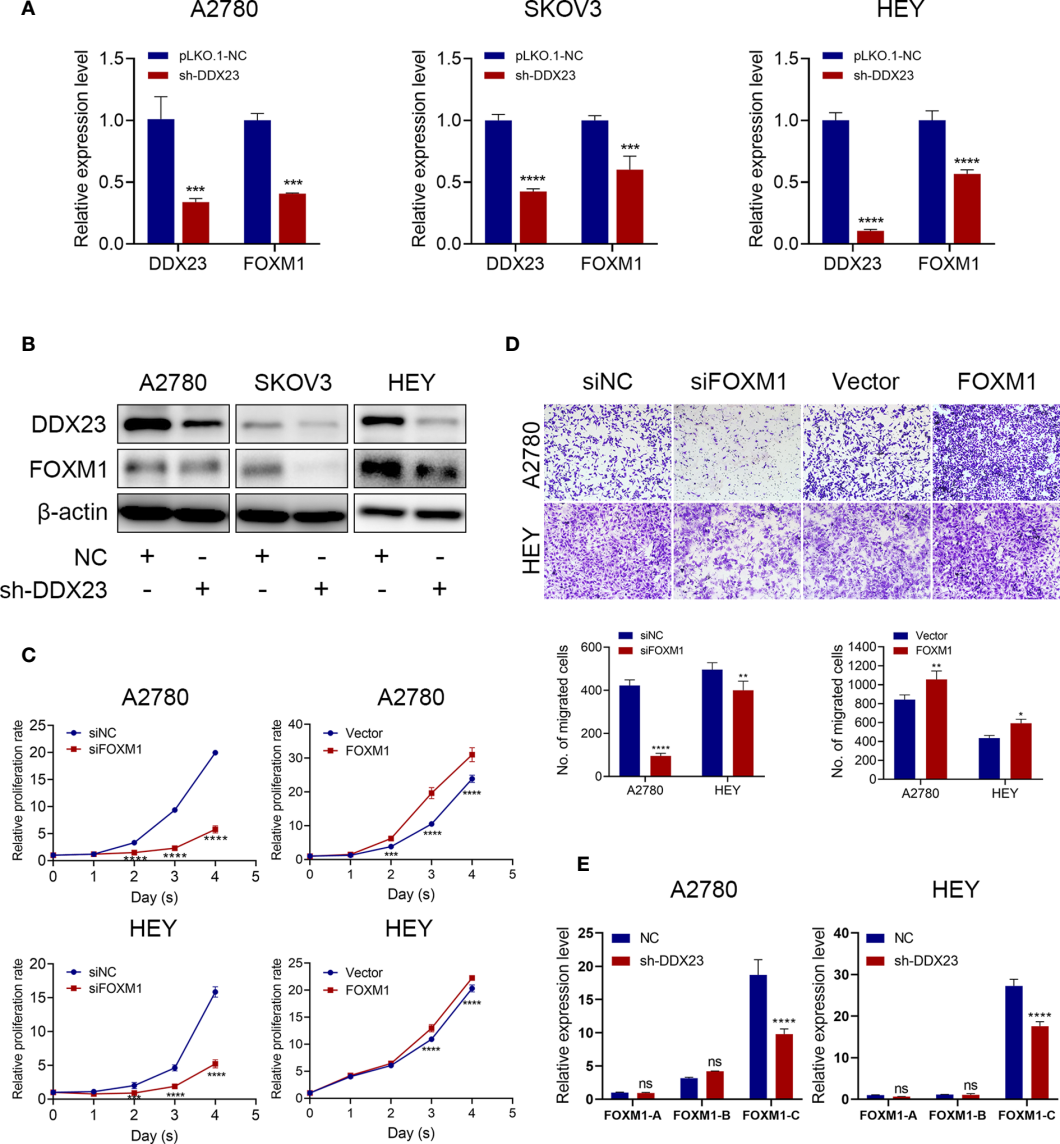
DDX23 regulated the production of the main oncogenic transcript of FOXM1. **(A, B)** The mRNA and protein levels of DDX23 and FOXM1 in ovarian cancer cells with or without DDX23 knockdown were measured by qRT-PCR **(A)** and western blotting **(B)**. **(C)** Representative MTT proliferation assays in A2780 and HEY cells with FOXM1 knockdown or overexpression. **(D)** Representative microscopic images (×10) of A2780 and HEY cells that penetrated through the Transwell chambers in migration assays. **(E)** Relative expression changes of different FOXM1 transcripts after DDX23 knockdown were analyzed in A2780 and HEY cells by qRT-PCR. Data are presented as mean ± SEM. ns, no significant, *P < 0.05, **P < 0.01, ***P < 0.001, ****P < 0.0001.

First, we measured FOXM1 expression after DDX23 knockdown and results showed that FOXM1 expression decreased at both mRNA and protein levels (**Figures 7A, B**). Moreover, we validated that FOXM1 silencing inhibited the proliferation and migration capacity of A2780 and HEY cells, while ectopic expression of FOXM1 enhanced their proliferation and migration potential (**Figures 7C, D**). It has been reported in glioma that DDX23 was an essential tool for miR-21 mature, revealing the powerful RNA processing function of DDX23 (20). However, whether FOXM1 mRNA processing was regulated by DDX23 remained unknown. The human FOXM1 gene consists of 10 exons, and the differential splicing of exons Va and VIIa produces three transcripts, FOXM1A, FOXM1B, and FOXM1C. Both FOXM1B and C have transcriptional activity, while FOXM1A does not due to the addition of exon VIIa in C-terminal transactivation domain (31, 37, 38). Subsequently, we measured the mRNA expression of FOXM1A, FOXM1B, and FOXM1C after DDX23 knockdown in A2780 and HEY cells by qRT-PCR. We found that FOXM1C expression was tens of times higher than the other two transcripts in ovarian cancer cells. FOXM1C expression was dramatically decreased after DDX23 knockdown, whereas FOXM1A and FOXM1B expression did not change significantly (**Figure 7E**). Therefore, these findings suggested that FOXM1 was a downstream target of DDX23. DDX23 was required for the mRNA processing of FOXM1. DDX23 silencing reduced the production of FOXM1C, the major oncogenic transcript of FOXM1 in ovarian cancer, thereby decreasing the FOXM1 protein expression and attenuating the malignant progression of ovarian cancer.

### FOXM1 Mediated DDX23-Driven Malignant Progression of Ovarian Cancer Cells

**Figure 8:**
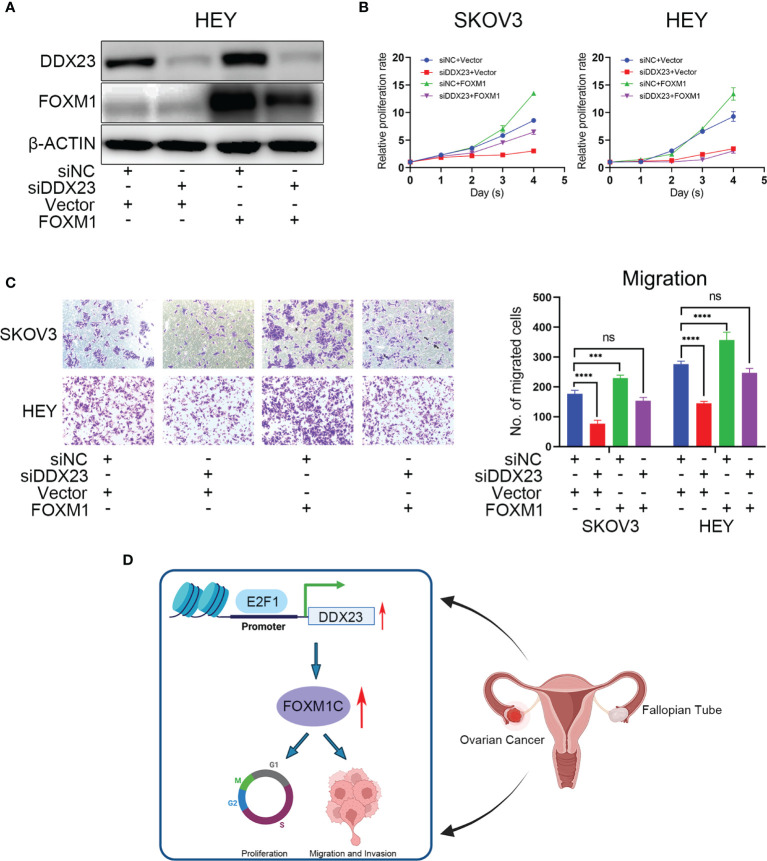
FOXM1 mediated DDX23-driven malignant progression of ovarian cancer cells. Control siRNAs or DDX23 siRNAs were co-transfected into ovarian cancer cells with PCMV-NC or PCMV-FOXM1 plasmids. **(A)** Western blotting analysis of DDX23 and FOXM1 expression in four rescue groups of HEY cells. **(B, C)** overexpression of FOXM1 partially restored the reduced cell proliferation **(B)** and migration **(C)** induced by DDX23 silencing. **(D)** Schematic diagram showing that DDX23 is transcriptionally activated by E2F1. DDX23 promotes ovarian cancer progression by regulating FOXM1C production. Data are presented as mean ± SEM. ns, no significant, ***P < 0.001, ****P < 0.0001.

In the foregoing sections, we demonstrated that FOXM1 mRNA and protein levels were decreased by DDX23 knockdown (**Figures 7A, B**). To determine whether DDX23 contribute to overall FOXM1 function, we performed rescue experiments by co-transfecting the ovarian cancer cells with DDX23 siRNA and FOXM1 plasmid, and examined the cell proliferation and migration. As expected, transfection of FOXM1 plasmid into HEY cells rescued the decreased FOXM1 protein levels caused by DDX23 knockdown (**Figure 8A**). In addition, FOXM1 overexpression enhanced the proliferation and migration capacity of SKOV3 and HEY cells. DDX23 knockdown significantly decreased cell proliferation and migration, whereas overexpression of FOXM1 partially restored the reduced cell proliferation and migration induced by DDX23 silencing (**Figures 8B, C**). Altogether, these results indicated that FOXM1 was a key executor in DDX23-induced malignant phenotype of ovarian cancer (**Figure 8D**).

## Discussion

The extreme malignancy of ovarian cancer is related to a variety of carcinogenic mechanisms, including mRNA processing dysregulation. Aberrant expression of splicing factors is implicated in tumor initiation and progression (39). Splicing factor SFPQ regulated alternative splicing of caspase-9 mRNA and was involved in ovarian cancer sensitivity to platinum (11). Our previous study showed that USP39 functioned as an oncogenic splicing factor in ovarian cancer through maintaining efficient splicing of HMGA2 (13). The DEAD-box RNA helicases are important members of the splicing factor family and they usually function as components of large multi-protein complexes and play essential roles in RNA processing including spliceosome biogenesis, miRNA biogenesis and splicing, which are crucial for cellular proliferation and transformation of tumorigenicity (40, 41). DDX23 expression was elevated in glioma patients and it had been strongly linked to the poor prognosis of glioma (20). DDX23 was also upregulated in hepatocellular carcinoma and correlated with advanced clinicopathological stages (42). However, its clinical significance and biological function in ovarian cancer have not been characterized to date. In our study, we first determined that DDX23 was overexpressed and significantly correlated with poor clinical outcomes in ovarian cancer. DDX23 expression was an independent high-risk factor closely associated with the OS of ovarian cancer patients. These results indicate that DDX23 can serve as an indicator of prognostic prediction in ovarian cancer patients.

To further explore the role of DDX23 in ovarian cancer, we performed relevant functional experiments *in vitro* and *in vivo*. DDX23 was previously reported to promote the invasion and proliferation of glioma cells (20). In hepatocellular carcinoma, SDC4/DDX23 axis played a crucial role in driving cell proliferation and migration (42). In our study, DDX23 silencing significantly impeded the proliferation of ovarian cancer cells through G1 phase arrest. The expression of associated cell cycle regulators also changed in DDX23-depleted ovarian cancer cells. In addition, loss of DDX23 also inhibited cell migration and invasion. These results highlight the pathogenic role of DDX23 in ovarian cancer.

However, the function mechanism of splicing factor DDX23 in ovarian cancer has not been elucidated. DEAD-box RNA helicase family proteins, including DDX23 (also known as Prp28), participate in the assembly of spliceosomes (43). For example, DDX23 is a mediator for switching the U1 snRNA/pre-mRNA 5’splice site (5’SS) base-pairing interaction (44). Prp28 mediates the transfer of the 5’SS from U1 snRNP to the U6 snRNA ACAGAGA sequence, which is an important prerequisite for the formation of the catalytic center of the spliceosome (45). DDX23 phosphorylation influences the formation of tri-snRNP and B complex (15, 46). Prp28’s ATPase is activated by the phosphorylated Npl3 to trigger specific conformational changes, which is essential for spliceosome remodeling (47). Therefore, DDX23 mainly perform their functions by processing the mRNA of downstream genes. Maintaining efficient splicing and promoting pre-mRNA maturation are important functions of splicing factors. For example, SF3B1 regulated KSR2 RNA maturation to promote endometrial cancer progression (48). hnRNPA2B1 improved the stability of Lin28B mRNA and enhanced malignant potential of ovarian cancer (49). We speculated that DDX23 might perform a similar function on FOXM1 mRNA.

Among the FOXM1 isoforms, FOXM1C is generally elevated and exerts oncogenic function. Kong et al. reported that FOXM1C was mainly expressed in pancreatic tumors and promoted the growth and motility of pancreatic cancer, whereas FOXM1A was commonly undetectable (31). The FOXM1C was predominantly overexpressed in esophageal cancer compared to the other FOXM1 isoforms and promoted its metastasis (50). Consistently, we overexpressed FOXM1C in ovarian cancer cells and observed that FOXM1C facilitated their proliferation and migration potential (**Figures 7C, D**). We also noted that the expression of FOXM1C in ovarian cancer cells was much higher than that of FOXM1A and FOXM1B. Moreover, FOXM1C expression was dramatically decreased after DDX23 knockdown, whereas FOXM1A and FOXM1B expression did not change significantly (**Figure 7E**). Because of the relative low expression of FOXM1A and FOXM1B in ovarian cancer cells, the FOXM1C expression presented the most significantly decrease after DDX23 knockdown. These findings suggest that DDX23 mainly regulates the generation of FOXM1C, the main oncogenic transcript of FOXM1, thereby regulating the malignant behavior of ovarian cancer. Further experiments are needed to study the specific mRNA processing mechanism of DDX23 on FOXM1.

Meanwhile, our study also investigated the DDX23 promoter region to predict potential TFs that might regulate the DDX23 upregulation observed in ovarian cancer. We found that the binding peak of E2F1 were enriched in the promoter region of DDX23 in HELA, MCF-7, U2OS and K652 cell lines. The E2F-family members have emerged as crucial transcriptional regulators of proliferation-promoting genes (51). The upregulation of E2Fs and their target genes has been linked with poor prognosis of various cancers, including breast and liver cancers (52, 53). E2F1, a member of the E2F-family activator subcategory, plays a crucial role in cancer cell proliferation, invasion, and apoptosis (54, 55). In ovarian cancer, low expression of E2F1 was reported to be correlated with favorable disease-free survival (DFS) and OS (56). In our study, we found that E2F1 knockdown decreased DDX23 expression at both the mRNA and protein levels. We subsequently confirmed that E2F1 could bind to the DDX23 promoter region directly and regulate DDX23 transcription in ovarian cancer cells. These results indicated that DDX23 was a direct transcriptional target of E2F1. Transcriptional activation of DDX23 by E2F1 in turn up-regulates DDX23 in ovarian cancer.

In summary, our study was the first to demonstrate that DDX23 was upregulated in ovarian cancer and was associated with poor clinical outcomes. High expression of DDX23 was involved in the malignant proliferation and aggressiveness of ovarian cancer cells by regulating FOXM1 mRNA processing. FOXM1 was a key executor in DDX23-induced malignant phenotype of ovarian cancer. Our study also revealed that DDX23 was transcriptionally activated by E2F1, contributing to the elevated expression of DDX23 in ovarian cancer.

Although we have confirmed that DDX23 is involved in the FOXM1 mRNA processing, the underlying mechanism of DDX23 regulating FOXM1 is still unclear. Whether DDX23 regulates FOXM1 mRNA processing directly or indirectly remains to be further studied. At present, small-molecule inhibitor targeting DDX23 is still unavailable. Therefore, tumor suppression experiments with the specific inhibitor cannot be completed *in vivo*, which might restrict its clinical transformation. However, our research provides a promising therapeutic target for precision treatment of ovarian cancer and also provides new insights into the important biological functions of splicing-related factors. With the development of molecular biology and the molecular structure analysis techniques, corresponding targeted drugs are expected to be developed and applied.

## Data Availability Statement

The raw data supporting the conclusions of this article will be made available by the authors, without undue reservation, to any qualified researcher. The RNA-seq data has been uploaded to GEO, and the accession number is GSE181078.

## Ethics Statement

The studies involving human participants were reviewed and approved by Ethics Committee of Shandong University. The patients/participants provided their written informed consent to participate in this study. The animal study was reviewed and approved by Shandong University Animal Care and Use Committee.

## Author Contributions

This study was conceived, designed, and interpreted by BK and KS. YL and KS were responsible for the comprehensive technical support. CZ and YL contributed to the data acquisition, analysis and interpretation. CZ, HW and CQ analyzed the clinical prognosis. CZ, JC, QW, HW, and XM collected the clinical samples. CZ was the major contributor in writing the manuscript. CZ and CQ contributed to the inspection of data and final manuscript. All authors contributed to the article and approved the submitted version.

## Funding

This work was financially supported by the National Natural Science Foundation of China (Nos. 81874107, 82072871, and 81902650), the Tai-Shan Scholar Program of Shandong Province (No. ts20070743), and the Key Research and Development Program of Shandong Province (No. 2019GSF108048).

## Conflict of Interest

The authors declare that the research was conducted in the absence of any commercial or financial relationships that could be construed as a potential conflict of interest.

## Publisher’s Note

All claims expressed in this article are solely those of the authors and do not necessarily represent those of their affiliated organizations, or those of the publisher, the editors and the reviewers. Any product that may be evaluated in this article, or claim that may be made by its manufacturer, is not guaranteed or endorsed by the publisher.

## Supplementary Material

Supplementary Figure S1 Relative mRNA expression of CDK9, E2F1, CBX5, SUZ12, and ATF1 in HGSOC and normal ovarian tissues using data from TCGA-GTEx. ns, no significant, ***P* < 0.01, *****P* < 0.0001.

Supplementary Figure S2
**(A, B)** The mRNA and protein levels of E2F1 in ovarian cancer cells with or without DDX23 knockdown were measured by qRT-PCR **(A)** and Western blotting **(B)**. ns, no significant, *****P* < 0.0001.

Supplementary Table 1 Sequences of siRNA, shRNA and primers used in this study.

Supplementary Table 2 Primary antibodies used in this study.

Supplementary Table 3 Genes positively related to DDX23 expression (TCGA U133 microarray) from cBioPortal database.

Supplementary Table 4 Transcription factors predicted to bind to the promoter of DDX23 from Cistrome Data Browser.

Supplementary Table 5 The potential E2F1 binding sites on the DDX23 promoter region from JASPAR online database.

Supplementary Table 6 Regular potential score of FOXM1 target genes from Cistrome Cancer.
