# Supplementary material for: Splicing Factor DDX23, Transcriptionally Activated by E2F1, Promotes Ovarian Cancer Progression by Regulating FOXM1
Source: Front Oncol. 2021 Dec 13;11:749144. doi: 10.3389/fonc.2021.749144 (PMC8710544; doi:10.3389/fonc.2021.749144)
Supplement: Supplementary Figure S1 — Relative mRNA expression of CDK9, E2F1, CBX5, SUZ12, and ATF1 in HGSOC and normal ovarian tissues using data from TCGA-GTEx. ns, no significant, **P < 0.01, ****P < 0.0001. [file DataSheet_1.pdf]

Supplementary Material

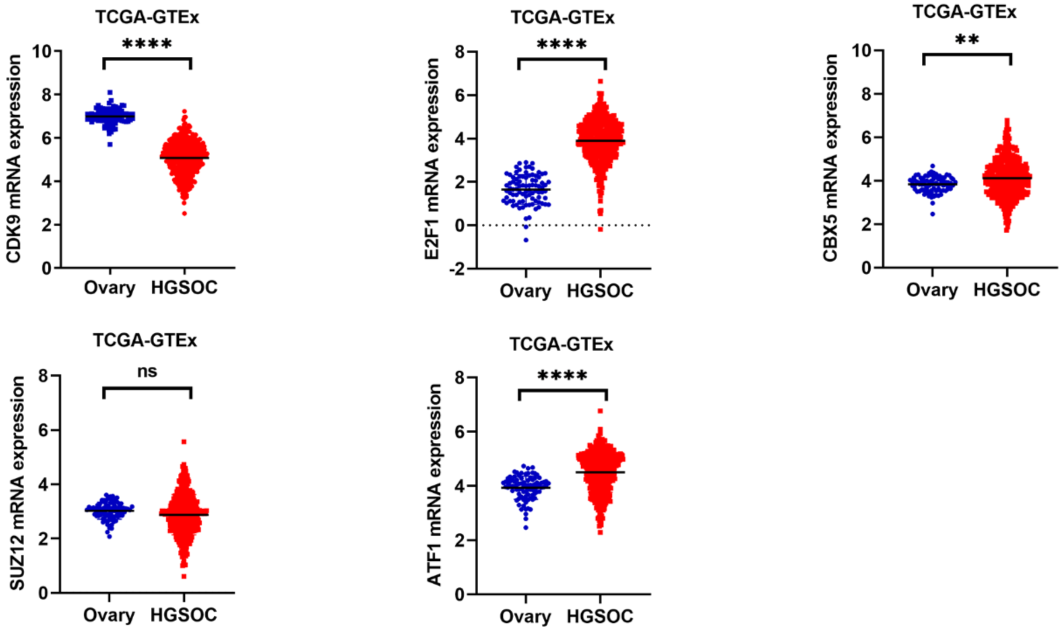

Supplementary Figure S1 | Relative mRNA expression of CDK9, E2F1, CBX5, SUZ12, and ATF1 in HGSOC and normal ovarian tissues using data from TCGA-GTEx. ns, no significant, **$P < 0.01$, ****$P < 0.0001$.

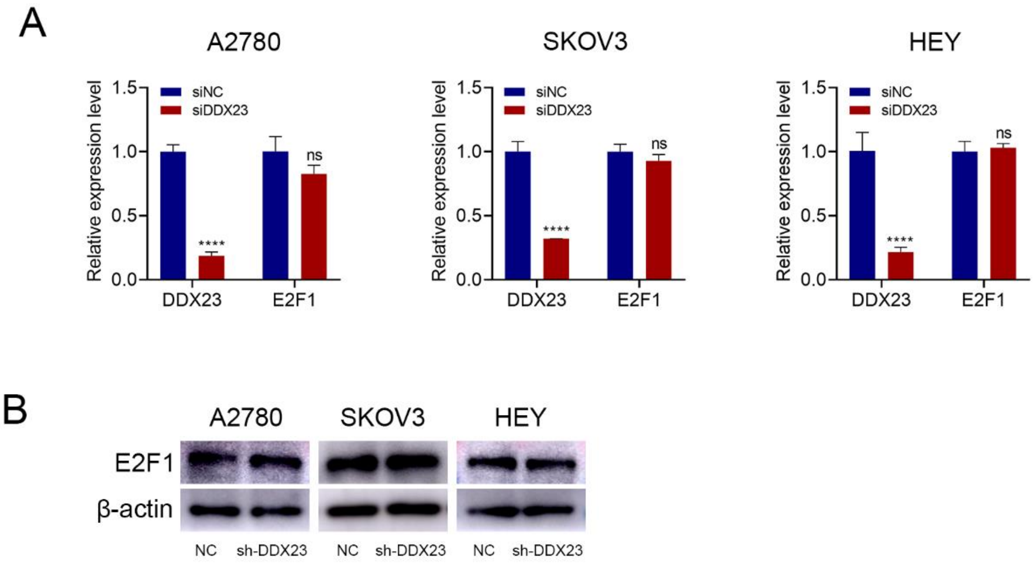

Supplementary Figure S2 | (A,B) The mRNA and protein levels of E2F1 in ovarian cancer cells with or without DDX23 knockdown were measured by qRT-PCR **(A)** and Western blotting **(B)**. ns, no significant, ****P < 0.0001.
