# Supplementary material for: Splicing Factor DDX23, Transcriptionally Activated by E2F1, Promotes Ovarian Cancer Progression by Regulating FOXM1
Source: Front Oncol. 2021 Dec 13;11:749144. doi: 10.3389/fonc.2021.749144 (PMC8710544; doi:10.3389/fonc.2021.749144)
Supplement: Supplementary Table 1 — Sequences of siRNA, shRNA and primers used in this study. [file Table_1.docx]

**Supplementary Table 1 |** Sequences of siRNA, shRNA and primers used in this study.

| method | name | Sequence (5 ′-3 ′) |
| --- | --- | --- |
| siRNA | negative control | UUCUCCGAACGUGUCACGUTT |
| siRNA | siDDX23 | GGAAACAGUUCCAAGACUUTT |
| siRNA | siE2F1 | CACTGAATCTGACCACCAA (target sequence) |
| siRNA | siFOXM1 | GCCCAACAGGAGTCTAATCAA (target sequence) |
| sh-RNA | sh-DDX23-F | CCGGAAGGAAACAGTTCCAAGACTTCTCGA GAAGTCTTGGAACTGTTTCCTTTTTTTG  GAAGTCTTGGAACTGTTTCCTTTTTTTG |
| sh-RNA | sh-DDX23-R | AATTCAAAAAAAGGAAACAGTTCCAAGACT TCTCGAGAAGTCTTGGAACTGTTTCCTT |
| qPCR | ACTB-F | CTCGCCTTTGCCGATCC |
| qPCR | ACTB-R | TCTCCATGTCGTCCCAGTTG |
| qPCR | DDX23-F | TAGAAAGCGGCATCGTTCAAG |
| qPCR | DDX23-R | AAGCTGGATCGTTTACGGTCC |
| qPCR | E2F1-F | GGACCTGGAAACTGACCATCAG |
| qPCR | E2F1-R | CAGTGAGGTCTCATAGCGTGAC |
| qPCR | ESPL1-F | ATCTCTGTCAGTCGGACCTGCA |
| qPCR | ESPL1-R | CAGGTGGACCTTCTTCACAGAG |
| qPCR | KIF14-F | GCACTTTCGGAACAAGCAAACCA |
| qPCR | KIF14-R | ATGTTGCTGGCAGCGGGACTAA |
| qPCR | TUBG1-F | CACTCAAGAGGCTGACGCAGAA |
| qPCR | TUBG1-R | GGTTGATCTGGGAGAAGGATGG |
| qPCR | KIF11-F | TGTTTGATGATCCCCGTAACAAG |
| qPCR | KIF11-R | CTGAGTGGGAACGACTAGAGT |
| qPCR | TOP2A-F | ACCATTGCAGCCTGTAAATGA |
| qPCR | TOP2A-R | GGGCGGAGCAAAATATGTTCC |
| qPCR | KIF23-F | AGTCAGCGAGAGCTAAGACAC |
| qPCR | KIF23-R | GGTTGAGTCTGTAGCCCTCAG |
| qPCR | TACC3-F | TCTTGGGAGCACTGGACATTCC |
| qPCR | TACC3-R | TCCAGGTCCTTCTGGCTGTACT |
| qPCR | CIT-F | AGCACAAGGCTGAGATTCTCGC |
| qPCR | CIT-R | CTCGTTCAGTCTCCAGCTTCTG |
| qPCR | PSRC1-F | CTCGAAAAGGGCTTCCAAGACC |
| qPCR | PSRC1-R | TGACAGGAAGATTTAGTCGCTGG |
| qPCR | NCAPH-F | GTCCTCGAAGACTTTCCTCAGA |
| qPCR | NCAPH-R | TGAAATGTCAATACTCCTGCTGG |
| qPCR | PLK1-F | GCACAGTGTCAATGCCTCCAAG |
| qPCR | PLK1-R | GCCGTACTTGTCCGAATAGTCC |
| qPCR | RBBP8-F | TGGCAGACAGTTTCTCCCAAGC |
| qPCR | RBBP8-R | GGCTCCACAAACGCTTTCTGCT |
| qPCR | MYBL2-F | CACCAGAAACGAGCCTGCCTTA |
| qPCR | MYBL2-R | CTCAGGTCACACCAAGCATCAG |
| qPCR | MCM4-R | CTTGCTTCAGCCTTGGCTCCAA |
| qPCR | MCM4-R | GTCGCCACACAGCAAGATGTTG |
| qPCR | CDC6-F | GGAGATGTTCGCAAAGCACTGG |
| qPCR | CDC6-R | GGAATCAGAGGCTCAGAAGGTG |
| qPCR | BUB1-F | GCTCTGTCAGCAGACTTCCTTC |
| qPCR | BUB1-R | CAGCAGATGTGAAGTCTCCTGG |
| qPCR | FOXM1-F | CATTAAGGAAACGCTGCCCA |
| qPCR | FOXM1-R | GGTTCTGAACTGAGGAGCCT |
| qPCR | FOXM1A-F | GAACATGACCATCAAAACCGAACTC |
| qPCR | FOXM1A-R | AAATTAAACAAGCTGGTGATGGGTG |
| qPCR | FOXM1B-F | GGACCAGGTGTTTAAGCAGCAG |
| qPCR | FOXM1B-R | CAATGCGGACTCGCTTGCTAT |
| qPCR | FOXM1C-F | TTGCCCGAGCACTTGGAATCA |
| qPCR | FOXM1C-R | TCCTCAGCTAGCAGCACCTTG |
| ChIP-PCR | DDX23-F | GACCAGGAAACGGGAAAGAT |
| ChIP-PCR | DDX23-R | GTTCATGCCCCTTCTCTGA |
