# Supplementary material for: Splicing Factor DDX23, Transcriptionally Activated by E2F1, Promotes Ovarian Cancer Progression by Regulating FOXM1
Source: Front Oncol. 2021 Dec 13;11:749144. doi: 10.3389/fonc.2021.749144 (PMC8710544; doi:10.3389/fonc.2021.749144)
Supplement: Supplementary Table 2 — Primary antibodies used in this study. [file Table_2.docx]

**Supplementary Table 2 |** Primary antibodies used in this study.

| Antibody | Company | Catalog number |
| --- | --- | --- |
| DDX23 | Proteintech | 10199-2-AP |
| DDX23 | Abcam | ab70459 |
| CCND1 | CST | #2978 |
| CDK4 | ABCAM | Ab108357 |
| P21 | ABCAM | #2947 |
| Ki-67 | CST | #9449 |
| E2F1 | CST | #3742 |
| FOXM1 | CST | #20459 |
| β-actin | Proteintech | 20536-1-AP |
